# An Unfortunate Case of Reactivation of Tuberculosis in a Postpartum Female

**DOI:** 10.7759/cureus.11775

**Published:** 2020-11-29

**Authors:** Azka Tasleem, Aqsa Mahmood, Anchit Bharat

**Affiliations:** 1 Internal Medicine, Ball Memorial Hospital, Muncie, USA; 2 Internal Medicine, Indiana University Health Ball Memorial Hospital, Muncie, USA

**Keywords:** postpartum tuberculosis, immune reconstitution, mycobacterium tuberculosis

## Abstract

Tuberculosis (TB) is a widely prevalent disease, especially in resource-limited settings. It poses a big burden to the community and is a significant cause of morbidity and mortality in pregnant females due to their immunosuppressed state. During pregnancy, the immune system is suppressed to prevent fetal rejection, and it gets reconstituted postpartum. During this reconstitution phase, reactivation of TB may occur, making it quintessential to test peripartum females for latent TB, especially those belonging to endemic regions. We describe an unfortunate case of reactivation of TB in a postpartum female from Central America.

## Introduction

Tuberculosis (TB) is an infectious disease caused by the bacteria - *Mycobacterium tuberculosis*. Due to its high affinity towards lung tissues, the most common manifestation is pulmonary TB [1]. The pathophysiology involves inhalation of the organism and its replication in smaller airways followed by engulfment by macrophages to form granulomas. Such granulomas in combination with lymph nodes form the Ghon's foci. The organism remains dormant in the granuloma until it gets favorable conditions for activation, e.g., immunosuppressive states [2]. Pregnant females are considered high risk for developing this disease, evidenced by a rate of 26.6 cases per one hundred thousand births in the United States from 2003 to 2011 [3].This risk is further increased in the postpartum state due to immune reconstitution. During pregnancy, Th1 pro-inflammatory cytokines such as gamma interferon, tumor necrosis factor-alpha, and interleukin (IL)-12 are suppressed, whereas Th2 and Th3 anti-inflammatory cytokines such as IL-10 and tumor growth factor-beta are enhanced [4]. These changes are mediated by maternal hormones like progesterone, cortisol, norepinephrine, and 1,25-dihydroxy vitamin D in order to prevent fetal rejection. It is reported that T lymphocytes’ response to purified protein derivative (PPD) of tuberculin is reduced in pregnant females [5]. *Mycobacterium tuberculosis* usually remains dormant in the granulomatous structure due to the effect of pro-inflammatory cytokines. During pregnancy, however, the bacteria might get reactivated due to a drop in these cytokine levels. The pathogenesis of active TB and symptom manifestation involves damage to the host caused by the organism as well as the host’s immune system. In pregnant females, the pro-inflammatory response and lymphocyte reactivity against the antigen are weak, which leads to reactivation of bacteria, however, clinical manifestations are not seen [6]. During the postpartum phase, due to immune reconstitution, the lymphocyte reactivity against PPD returns to normal and pro-inflammatory cytokines return to their physiologic levels or even higher. This lymphocyte activity generates a strong immune response against PPD and causes damage to the host leading to clinical signs and symptoms [7]. This phenomenon can be explained by the theory of damage-response framework, which endorses that the pathogenesis of a disease depends not only on the microorganism but also on the host’s immune system [8]. This framework divides microorganisms into different classes depending on their interaction with the hosts and timing of peak disease. In accordance with this framework, *M. tuberculosis* is considered as an organism that causes maximal damage at the peak of immune response, thus when there is a rapid increase in immune response during postpartum period, the risk for clinical disease increases. The concept of postpartum reactivation of tuberculosis (TB) is an example of how the immune system can help eradicate disease on one hand, and on the other, cause damage to the host before disease resolution.

## Case presentation

**Figure 1:**
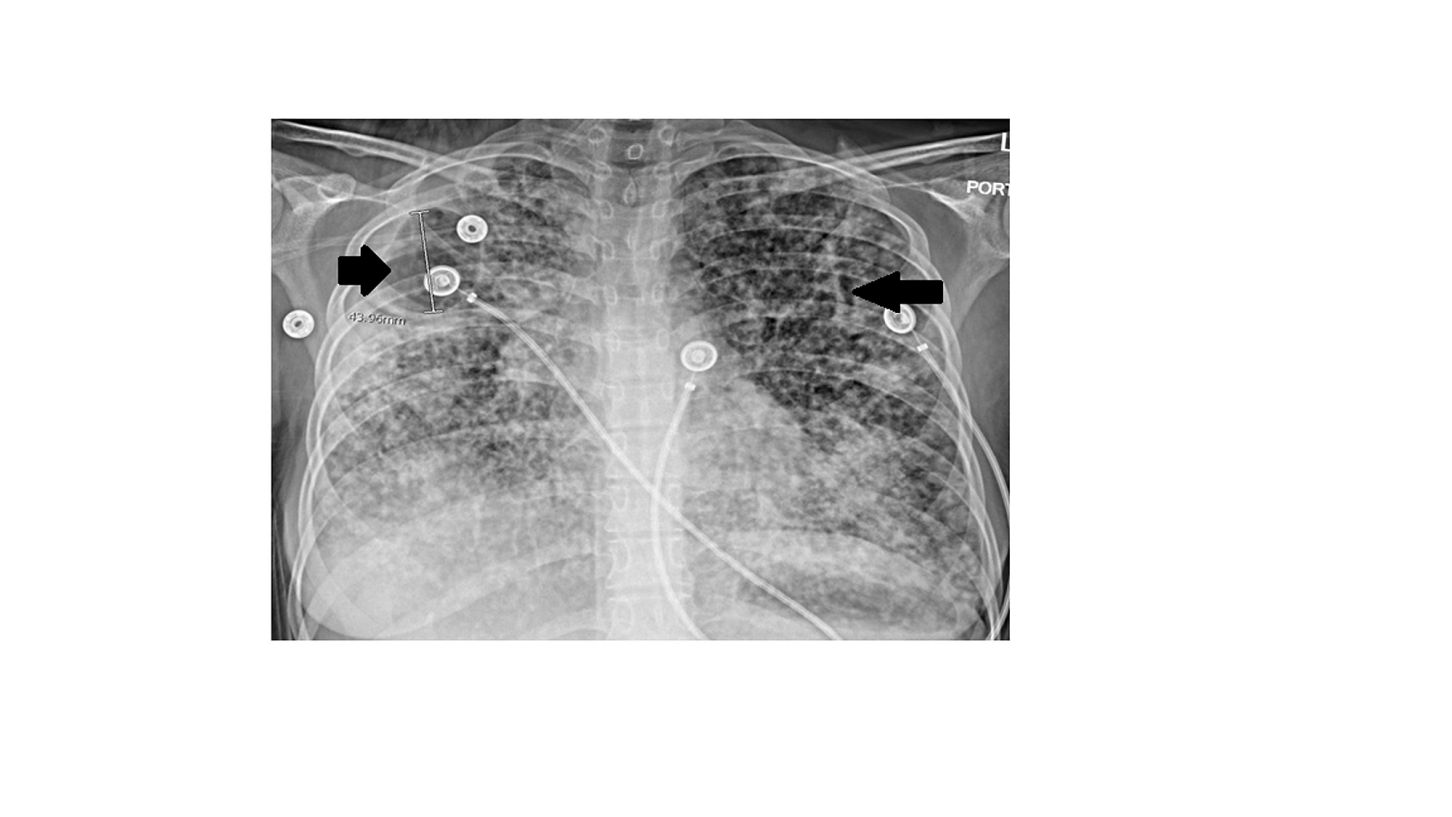
Radiograph PA view showing bilateral diffuse airspace disease and apical cavitary lesion. PA, posteroanterior

A 21-year-old female originally from Guatemala, with a past medical history significant for recent childbirth (three months prior), presented to the outside hospital ED with fever, cough, and shortness of breath of two weeks duration, preceded by generalized malaise for two months. Chest X-ray, done in ED, showed extensive opacities as follows (Figure 1).

**Figure 2:**
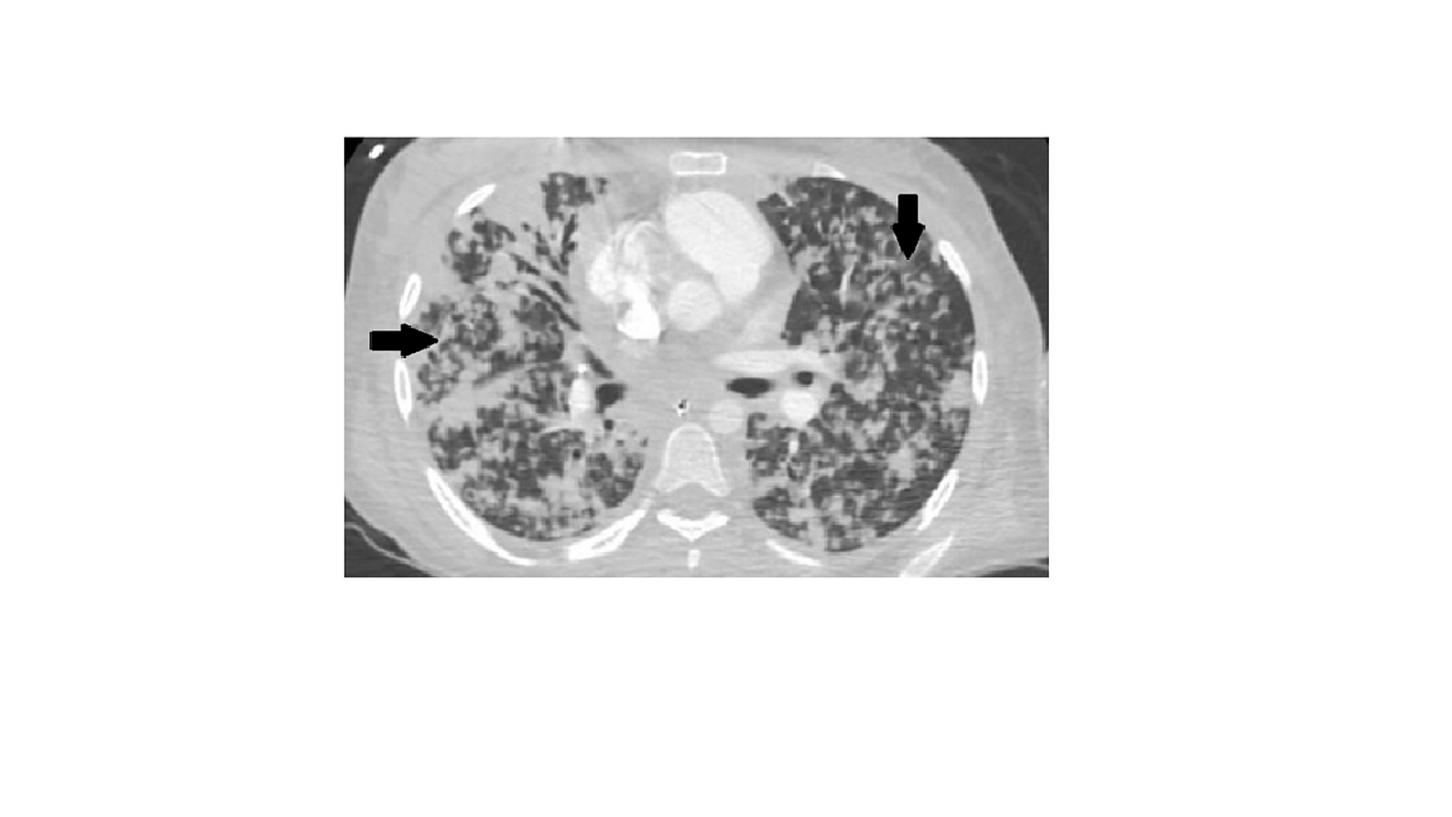
CTA chest transverse view showing diffuse nodular opacities as indicated by arrows. CTA, CT angiogram

**Figure 3:**
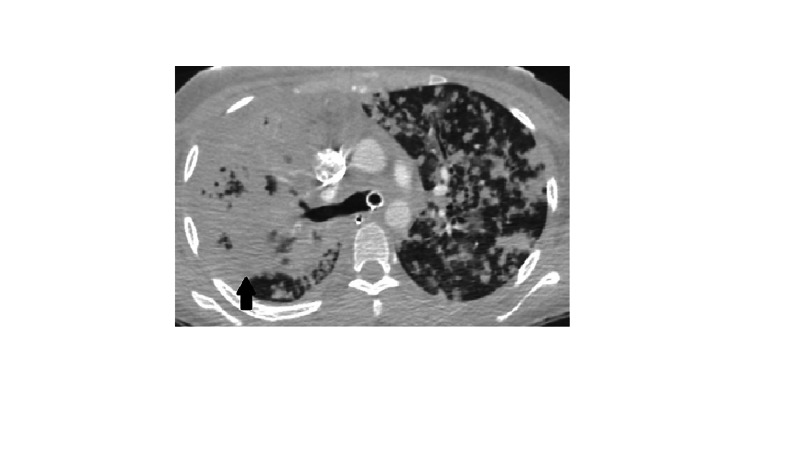
CTA chest transverse view showing confluent areas of opacification as indicated by arrows. CTA, CT angiogram

**Figure 4:**
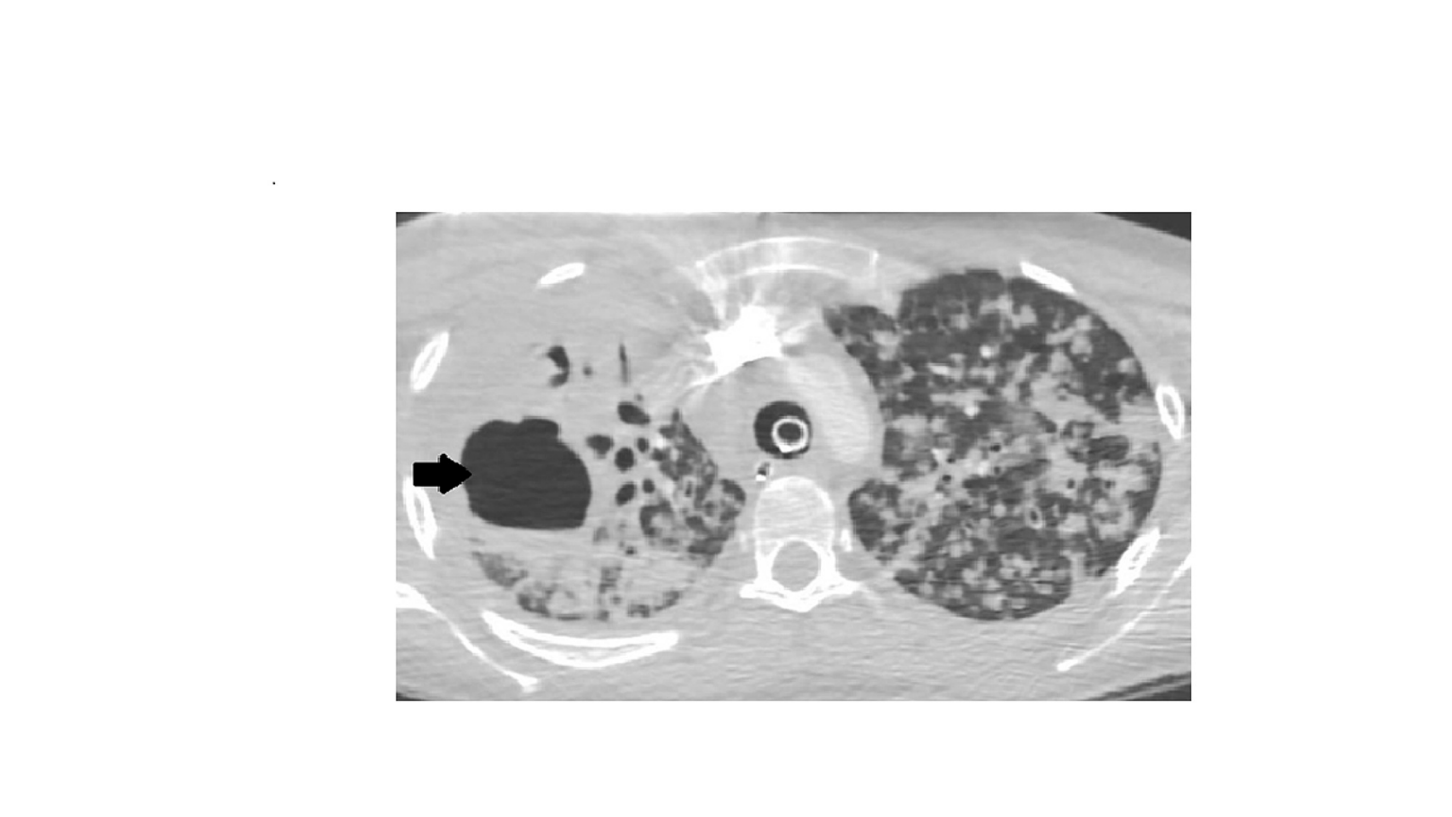
CTA chest transverse view showing a cavitary lesion, measuring 5.1 cm in maximum dimension with surrounding peribronchial thickening as indicated by arrows. CTA, CT angiogram

In the ED, the patient was given IV fluids, azithromycin, cefepime and was placed on bi-level positive pressure ventilation (BiPAP). The patient was transferred to our ICU from the outside hospital to escalate care. During transport, she became hypotensive, her oxygen saturation dropped, and mechanical ventilation was attempted after placing an endotracheal tube. Soon after rapid sequence intubation (RSI) with 100 mg ketamine and 100 mg succinylcholine, the patient became bradycardic (20-30 bpm) and blood pressure dropped further (mean arterial pressure, MAP < 65). Emergency Medical Services (EMS) started the patient on dopamine 10 mcg/kg/min without response. Soon after, the patient became pulseless and cardiopulmonary resuscitation (CPR) was initiated. Initial rhythm reported was ventricular fibrillation for which defibrillation was attempted (360 J). On arrival to ED, the initial rhythm was asystole. The patient received multiple rounds of CPR and epinephrine, before return of spontaneous circulation (ROSC) was achieved. History was otherwise limited due to the patient's altered mental status. Physical examination was remarkable for a Glasgow coma scale (GCS) of 3, coarse bilateral breath sounds with end-inspiratory and end-expiratory crackles. CT head without IV contrast done in the ED showed no evidence of intracerebral bleeding or mass lesions. Above Chest X-ray (CXR) findings were confirmed with a CT angiogram (CTA) of the chest which showed a cavitary lesion in the right upper lobe with peribronchial thickening and bilateral diffuse tree-in-bud nodular opacities. No evidence of pulmonary embolism was seen (Figures 2-4).

The patient’s labs were significant for elevated leukocyte count (16.8 k/cumm), mild hyponatremia (129 mmol/L), elevated D-dimer (3333 ng/mL DDU), elevated alkaline phosphatase (506 units/L), transaminitis with aspartate aminotransferase (206 units/L), alanine aminotransferase (55 units/L), elevated lactate (9.9 mmol/L), and procalcitonin (82.09 ng/mL). The patient was started on vancomycin and piperacillin tazobactam. On further evaluation, testing for urine streptococcal antigen, urine legionella antigen, methicillin resistant staphylococcus aureus (MRSA) polymerase chain reaction, and severe acute respiratory syndrome coronavirus 2 (SARS-CoV-2) was negative. Serum analysis for coccidia, histoplasma, blastomyces and cryptococcal antigen, antinuclear (ANA), anti-double-stranded, hepatitis A, B, E antibodies and human immunodeficiency virus was negative. Blood cultures were negative. Although she had a positive (405 pg/mL) fungitell, only a few candida and geotrichum species were isolated on culture. These were considered part of normal respiratory flora.

During her hospital course, her respiratory cultures were positive for acid fast bacillus (AFB). Infectious disease specialist was consulted and the patient was started on isoniazid, rifampin, pyrazinamide, and ethambutol for pulmonary TB treatment. Further confirmation with bronchoscopy was done. The bronchoalveolar lavage fluid (BAL) was clear and consisted of a high neutrophil count (93%), normal lymphocyte count (2%), and low monocyte/macrophage count (5%). BAL analysis for bacterial pathogens like *Pseudomonas*, *Hemophilus influenzae*, *Staphylococcus aureus*, *Streptococcus agalactiae*, *Streptococcus pneumonia*, and *Pneumocystis* was negative. BAL fluid cultures were negative except for AFB, a few candida and geotrichum species. The patient continued to be unresponsive, and a repeat CT head was done which showed possible cerebral edema and tiny area of hyperdensity in frontal lobe. Subsequently, MRI revealed severe anoxic brain injury with cerebral edema and small subarachnoid hemorrhage. In the setting of pulmonary TB and encephalopathy, lumbar puncture was done to rule out tuberculous meningitis. Cerebrospinal fluid (CSF) analysis showed a high neutrophil count (95%), low lymphocyte count (1%), and normal monocyte count (4%). The composition included low glucose (39 mg/dL), high protein (47 mg/dL), and high lactate dehydrogenase (LDH) (893 units/L) levels. Although CSF cultures were negative, due to high suspicion for tuberculous meningitis, steroids were started. Neurology was consulted and recommended an electroencephalogram which showed diffuse slow 2 and 2-½ cycles per second seen predominantly in the posterior regions, suggesting cerebral dysfunction. Palliative care was consulted due to sustained neurological unresponsiveness and a poor outlook for recovery. After an extended family discussion, withdrawal of care was done.

## Discussion

In highly endemic areas, TB is one of the leading causes of mortality in females during reproductive years [9]. Moreover, the rate of TB is higher in postpartum than nonpregnant females [10]. Maternal immune system is suppressed in pregnancy to prevent an immune response against the fetus [11]. Research shows that immune reconstitution postpartum plays a role in the reactivation of infections such as TB. TB predisposes pregnant females to complications like low birth weight babies, spontaneous abortions, etc [12]. In this study, we discuss a young female who was three months postpartum and presented with two-weeks of cough and shortness of breath. An extensive workup was consistent with a diagnosis of pulmonary TB.

Our patient was not tested prepartum, despite being from a TB endemic area. She was HIV negative and had no recent exposure to a TB patient. Through this study, we aim to bring up the question - how extensively the clinicians should work up latent TB patients during pregnancy to reduce reactivation of TB postpartum? Untested latent cases can progress to active infection and are at risk of developing maternal and fetal complications, including death and pose a significant financial burden on healthcare resources. In the United States, the cost of hospitalization for TB is estimated to be 38.4 million dollars from 2010 to 2014 [13]. Hence, better preventative strategies, earlier testing, and prompt initiation of antituberculous therapy are warranted to provide the best care to our patients. 

Moreover, our patient’s CSF sample was predominantly neutrophilic, and the cultures were negative. Research has shown that patients with tuberculous meningitis can develop neutrophilic pleocytosis initially before the CSF acquires lymphocytic composition [14]. CSF cultures are deemed less sensitive for tuberculous meningitis. In this light, we proceeded with empiric antibiotic therapy and steroids despite negative CSF cultures [15].

We encourage our readers to have a low threshold of having TB as a differential diagnosis for a patient presenting with respiratory and infectious symptoms in a postpartum state. Although theory of postpartum immune reconstitution is considered the cause of reactivation, further research is required to explain the precise mechanism and if any measures can prevent it. Our case report emphasizes the significance of prompt diagnosis and treatment of latent TB during pregnancy to prevent adverse outcomes. More studies are required to document such cases in order to have a better understanding of incidence of reactivation in pregnancy.

## Conclusions

Tuberculosis is a life-threatening though preventable disease. It has affected millions of people worldwide and despite constant efforts to limit its spread, there are many areas with a high incidence of TB. In the United States, reactivation of latent TB is a known phenomenon postpartum. Hence, pregnant females need to be tested to diagnose TB in its latent state to prevent conversion to an active form and avoid the complications of active infection at both individual and community level.
